# Fault Root Cause Analysis Based on Liang–Kleeman Information Flow and Graphical Lasso

**DOI:** 10.3390/e27020213

**Published:** 2025-02-19

**Authors:** Xiangdong Liu, Jie Liu, Xiaohua Yang, Zhiqiang Wu, Ying Wei, Zhuoran Xu, Juan Wen

**Affiliations:** 1 School of Computer Science, University of South China, Hengyang 421001, China; 20222008110478@stu.usc.edu.cn (X.L.); xiaohua1963@usc.edu.cn (X.Y.); xuzhuoran@usc.edu.cn (Z.X.); wenjuan@usc.edu.cn (J.W.); 2 National Key Laboratory of Nuclear Reactor Technology, Nuclear Power Institute of China, Chengdu 610213, China; greyshark97@139.com (Z.W.); jennywy0987@139.com (Y.W.)

**Keywords:** root cause analysis, Liang–Kleeman information flow, graphical lasso

## Abstract

Root cause analysis is used to find the specific fault location and cause of a fault during system fault diagnosis. It is an important step in fault diagnosis. The root cause analysis method based on causality starts from the origin of the causal connection between transactions and infers the location and cause of the mechanism failure by analyzing the causal impact of variables between systems, which has methodological advantages. Causal analysis methods based on transfer entropy are proven to have biases in calculation results, so there is a phenomenon of calculating false causal relationships, which leads to the problem of insufficient accuracy in root cause analysis. Liang–Kleeman information flow (LKIF) is a kind of information entropy that can effectively carry out causal inference, which can avoid obtaining wrong causal relationships. We propose a root cause analysis method that combines graphical lasso and information flow. In view of the large amount of redundant information in industrial data due to the coupling effect of industrial systems, graphical lasso (Glasso) is a high-precision dimensionality reduction method suitable for large-scale and high-dimensional datasets. To ensure the timeliness of root cause analysis, graphical lasso uses dimensionality reduction of the data. Then, LKIF is used to calculate the information flow intensity of each relevant variable, infer the causal relationship between the variable pairs, and trace the root cause of the fault. On the Tennessee Eastman simulation platform, root cause analysis was performed on all faults, and two root cause analysis solutions, transfer entropy and information flow, were compared. Experimental results show that the LKIF–Glasso method can effectively detect the root cause of faults and display the propagation of faults throughout the process. It further shows that information flow has a better effect in root cause analysis than transfer entropy. And through the root cause analysis of the step failure of the stripper, the reason why information flow is superior to transfer entropy is explained in detail.

## 1. Introduction

In industrial systems, ensuring stable and efficient operations is crucial. Effectively and promptly detecting and locating the source of faults when they occur is a crucial issue. The critical step in addressing this challenge is to conduct a fault root cause analysis [1]. Root cause analysis involves analyzing the characteristics of the system’s fault symptoms and tracing them back to the faulty components within the system. Root cause tracing is the reverse process of fault propagation. Therefore, it is essential to clarify that fault propagation occurs when the fundamental cause of the device exhibits abnormal conditions. The close interconnections in the production process and the interactions between equipment lead to the transfer of matter, energy, and information in the control loop [2].

Modern industrial systems are control systems, and the control relationships between state variables in control systems belong to a type of causal relationship. Causal analysis methods can infer the root causes of fault symptoms by analyzing the causal influences between process states, and the causal analysis method can also show good performance for unknown faults. The causal influences between state variables can be measured by changes in entropy values [3]. Currently, there are mainly two information entropy-based causal analysis methods: transfer entropy method (TE) and Liang–Kleeman information flow method. The transfer entropy method is more effective in causal relationship analysis in nonlinear industrial processes than previous methods that relied on linear assumptions between variables. It has been applied not only in fault tracing but also in fault detection and fault isolation. However, the transfer entropy method may yield biased results and calculate false causal relationships [4].

As a new means of exploring causal relationships, the Liang–Kleeman information flow method has been applied in the field of fault diagnosis with good results [5]. The Liang–Kleeman information flow method has advantages in handling industrial nonlinear systems and time series data. It can differentiate between direct causal relationships and indirect causal relationships, making it a powerful tool to address false causal issues.

Due to the complexity of modern industrial control systems, which involve thousands of process variables, faults and their characteristics only concern a small portion of these variables. Therefore, in relation to the entire system, fault information within industrial systems can be considered sparse. To prevent redundant non-fault data from affecting the results of root cause analysis, it is necessary to first consider dimensionality reduction of industrial process data. Assuming that industrial fault information is sparse, it is appropriate to employ sparse methods for dimensionality reduction and extraction of fault information from industrial systems. The selection of sparse methods needs to take into account the complexity of the physical and chemical processes in industrial systems, which can introduce nonlinearity in the data, requiring sparse methods to have great adaptability to nonlinear data [6].

Jerome Friedman and others proposed a statistical method for estimating relationships between high-dimensional variables, known as graphical lasso [7]. The graphical lasso combines the concepts of lasso penalties and graph models. It penalizes values close to zero in the estimated inverse covariance matrix to zero, resulting in a sparse inverse covariance matrix. Non-zero values in the matrix indicate correlations between variables, while zero values indicate no interactions between variables, thus constructing a graphical representation of variable relationships. The lasso penalty mechanism helps the model adapt to nonlinear data by forcing some edge weights to shrink to zero, thereby reducing the model’s sensitivity to outliers and nonlinear data.

This study proposes a root cause analysis method that combines time series analysis with graph techniques. Root cause analysis requires correctly examining causal relationships between variables to explore root causes. Interpretability is crucial in root cause analysis. Clear explanations ensure that problems are accurately and effectively resolved rather than merely focusing on fault symptom variables. Graphical lasso introduces a graph model based on extracting variable relationships under the assumption of conditional independence. The sparse graph filters out numerous irrelevant variable pairs and visually displays the relationships between variables. This aids in fault tracing and enhances interpretability. Building upon the sparse graph computed by graphical lasso, Liang–Kleeman information flow (LKIF) calculates causal relationships for highly correlated variable pairs. This method improves interpretability by analyzing fault propagation paths based on the sparse graph obtained through graphical lasso while also preventing the occurrence of spurious causality resulting from the use of conditional independence measures in graphical lasso. Throughout this paper, this method is referred to as the LKIF–Glasso method, combining time series analysis, graphical lasso, and Liang–Kleeman information flow to enhance the interpretability and effectiveness of root cause analysis.

The information flow–Glasso method proposed in this paper makes the following two contributions:

1. It introduces a novel approach that combines time series analysis techniques with graph methods. This new method integrates the interpretability advantages of graph methods with the causal inference capabilities of time series methods. By merging these two approaches, the method aims to provide a comprehensive analysis framework that leverages the strengths of both methodologies.
2. The method attempts to address the issue of spurious causality in current root cause analysis methods by utilizing the Liang–Kleeman information flow (LKIF) technique, achieving promising results in mitigating false causal relationships.

The provided text describes the application of different causal inference methods, including information flows and common causal reasoning methods, to a widely used Tennessee Eastman Process(TEP) case. The performance of these methods was compared, focusing on TEP step fault 7. A step fault is characterized by a sudden significant increase or decrease in certain parameter values, resulting in a large fault magnitude and relatively low difficulty in fault detection. However, in some cases, due to the response of the control system, the process may quickly be adjusted back to an operating point close to normal conditions, leading operators to mistakenly believe that the fault has been eliminated. Nevertheless, due to the influence of the control system response, the relationships between process variables change, leading to a potential misdiagnosis [8]. The analysis results indicate that the LKIF–Glasso method successfully identified the source of the step fault and ultimately completed the trace-back of all TEP faults.

## 2. Related Work

When industrial systems experience faults, the interconnected components and numerous control loops within them cause faults to propagate rapidly along physical and information coupling pathways. The occurrence of numerous abnormal alarms for process variables can mask the true source of the fault. Root cause analysis methods need to accurately identify the fault-related components from these alarms to trace back to the origin of the fault [1]. Existing root cause analysis methods can be broadly categorized into two types: rule-based modeling methods and data-driven digital methods [9]. Among these methods, data-driven approaches have gained significant traction in research.

### 2.1. Related Work on Dimensionality Reduction

One commonly used type of dimensionality reduction method in fault diagnosis is multivariate statistical methods, including principal component analysis (PCA), independent component analysis (ICA), partial least squares (PLS), and non-negative matrix factorization (NMF). However, industrial data typically involve complex dynamic relationships and are fundamentally nonlinear. PCA and PLS have limitations in handling the nonlinearity of data, as they reduce the dimensionality of variables under a linear assumption [9]. Additionally, isolating faults using these methods can be challenging due to the simplification of various features of variables into lower dimensions.

Researchers have proposed their solutions to address this issue, such as using kernel PCA [10], generalized PLS [11], and ICA [12]. Kernel PCA combines the kernel space in reducing the dimensionality of the variable space, allowing for a more rigorous consideration of nonlinear relationships between variables in the kernel space. Generalized PLS adapts to various data distributions and relationships by introducing nonlinear link functions and distribution assumptions. ICA assumes that non-Gaussian source signals are mutually independent, separating signals or data into independent variables. While these methods help develop more suitable root cause analysis methods for nonlinear data, they may not effectively provide fault propagation paths [6].

Considering that fault information relative to the entire process data in industrial data is sparse, sparsification methods are also applied in dimensionality reduction. JIANG J proposed a method based on Lasso regression to establish a fault diagnosis model [13]. ZHENG K introduced a generalized contribution graph framework combined with group lasso and demonstrated the superiority of the proposed method in extracting weak fault characteristics [14]. Lasso regression does not strictly require linear distribution of data, and it can provide useful results even when data distribution exhibits nonlinear characteristics. However, these two methods do not analyze fault propagation paths, thereby lacking the ability to precisely locate root cause variables.

### 2.2. Related Work on Fault Tracing

There are currently two main solutions for root cause tracing. The first solution is to learn the historical data characteristics of the industrial system under normal and abnormal working conditions, and at the same time identify the machine status based on the learned characteristics, so as to achieve the purpose of tracing the cause. The second option is to clearly reveal the interaction between variables through causality analysis and gradually derive from the fault variable to the root cause variable based on the interaction. However, when faults are separated from the training data, methods based on historical data features inevitably have the limitation of showing unreliable performance in unknown situations. The causal relationship analysis method does not require training data and can still maintain good performance when unknown faults occur.

Typical methods for detecting causal relationships from process data include Granger causality, convergent cross-mapping, and transitive entropy. Although the above method is an effective causal analysis method, there is a problem of false causality in certain circumstances. Granger causality is a statistical testing method that is widely used due to its advantages in interpretability and implementability [15]. Although Granger causality can effectively use monitoring data to infer causality between components, it needs to meet the premise that the relationship between variables is linear. For industrial systems with complex coupling relationships and numerous nonlinear factors, Granger causality is difficult to accurately discover the causal connections that exist. Therefore, there are many improved methods to extend Granger causality to nonlinear multivariable processes [16].

Convergent cross-mapping is a new causal relationship modeling method based on dynamic models that can identify causal relationships in nonlinear systems [17]. However, when the causal relationship between variables is too strong, convergent cross mapping has difficulty in distinguishing between unidirectional causality and bidirectional causality. Ye et al. studied this issue and made up for the shortcoming of CCM that cannot distinguish false causality [18].

TE is based on the idea of information entropy and performs causal inference by calculating the degree to which uncertainty is reduced by one variable under the influence of another variable. Its main advantage is that it is suitable for determining whether there is a linear relationship between variables and whether there is a nonlinear relationship between variables [3]. However, the value of entropy transfer depends on the autoregressive coefficient of the dynamic system, resulting in biased results [4]. This bias causes TE to calculate spurious causal relationships. There have been many improvements in the TE algorithm in fault causal analysis of complex industrial processes [19,20,21]. However, they are often purely dedicated to improving calculation efficiency or optimizing parameters, ignoring the characteristic changes of fault data in different states. The problem of spurious causation of TE remains to be solved.

## 3. Root Cause Analysis Method Based on LKIF–Glasso

### 3.1. Root Cause Analysis Method Based on Graphic Lasso

When the state of an industrial system deviates from the expected values during operation, it indicates a malfunction in the system. Since the operation of the entire industrial system is based on production process requirements and follows control principles, the control system’s real-time transfer of energy and information is achieved by coupling the control process. This inevitably leads to the propagation and development of faults. Therefore, the appearance of fault signs at alarm points may not necessarily be the root cause of the failure. However, the system’s operating state is described through system variables, parameters, and other physical quantities. Due to the complexity of modern industrial systems, the system variables used to describe the operating state exhibit high-dimensional characteristics. When conducting root cause analysis, the interdependence between variables is influenced by the complex structure of industrial systems. Variables not influenced by fundamental causes also exhibit high correlation, affecting the efficiency and accuracy of fault analysis [22].

Therefore, dimensionality reduction must be considered first for root cause analysis of complex industrial systems. However, random disturbances and equipment degradation in industrial systems result in nonlinear characteristics in industrial data. This leads to the extraction of incorrect fault features by dimensionality reduction methods based on linear assumptions. Additionally, during the fault propagation process, the correlations between process variables may change with the response of numerous control loops, leading to new relationships. Variable correlations must be considered to avoid missing critical fault propagation paths and resulting in incorrect root cause conclusions. Addressing these issues requires the application of a dimensionality reduction method capable of extracting variable relationships.

Fault tracing is the reverse process of fault propagation. After extracting fault-related variables based on dimensionality reduction methods, it is necessary to analyze the mechanisms of influence between variables to ultimately pinpoint the root cause. However, based on the definition of correlation, the variable correlations estimated by dimensionality reduction methods only indicate the phenomenon of simultaneous changes between two variables. Refining these correlations into variable relationships with analytical and reasoning capabilities is essential. Due to the feedback control effect of industrial systems, process monitoring variables exhibit dynamic characteristics related to time (temporal characteristics). Variables possess both temporal and correlational aspects, satisfying the conditions for causality. Therefore, considering causal relationship analysis to infer the mechanisms of influence between variables is warranted.

Based on the needs of high-dimensional variable dimensionality reduction and variable relationship inference, root cause analysis should be divided into two steps. The first step is to use the dimensionality reduction method to reduce the dimensionality of high-dimensional variables. Dimensionality reduction requires attention to two issues, data nonlinearity and latent variable relationships, so as to avoid affecting the accuracy of fault traceability. In the second step, based on the connection relationship extracted by the dimensionality reduction method, the causal analysis method determines the causal relationship between the two time series variables to confirm whether there is a propagation relationship among the fault-related variables and the direction of local propagation of the fault, and identify the fault development path, and thereby trace the source of the fault and complete the root cause analysis of the fault.

Building upon these concepts, LEE, Hodong, and others have proposed a root cause analysis method that combines graphical lasso and transfer entropy [23]. Addressing the high dimensionality of industrial monitoring data, they employ a graphical lasso to filter fault-related variables and construct an undirected graph structure of relationships between process variables, reducing interference factors when analyzing primary relationships. Subsequently, transfer entropy is introduced to transform variable correlations into directional causal relationships. Finally, based on significant causal relationships calculated by transfer entropy, propagation paths are identified to trace the fault back to its source and complete fault tracing.

Graph lasso is a fast algorithm for estimating the inverse covariance matrix, which obtains a sparse representation of the inverse covariance matrix by adding the L1 regularization term and has the ability to infer variable relationships in high-dimensional data [24,25].

The Graphical lasso algorithm first assumes that the p-dimensional random variable x obeys Gaussian distribution, and defines the mean value of x as μ and the covariance matrix as ∑. If there are m observed samples, the logarithmic likelihood function can be written as follows:

(1) $$logL(\mu ,\sum )=C+\frac{m}{2}logdet(\sum^{-1})-\frac{1}{2}\sum_{i=1}^{m}{\left(x^{\left(i\right)}-\mu \right)}^{T}\sum^{-1}(x^{\left(i\right)}-\mu ),$$

Where C is a constant term.

Since this constant term does not depend on μ and ∑, the constant term C does not affect the estimation of parameters when the log-likelihood function is maximized, so the constant term C can be ignored. When logL(μ,∑) reaches the maximum value, the optimal covariance matrix or accuracy matrix can be obtained. Fault information is generally sparse, so the L1-Penalty term $\lambda \sum_{j\;\neq \;k}\left|\sum_{jk}^{-1}\right|\;$ is added to enhance the sparsity of the accuracy matrix. The accuracy matrix of the required solution is expressed as

(2) $${\hat{\sum }}^{-1}={argmin}_{\sum^{-1}\geq 0}(\frac{1}{m}\sum_{i=1}^{m}{\left(x^{\left(i\right)}-\mu \right)}^{T}\sum^{-1}(x^{\left(i\right)}-\mu )-logdet(\sum^{-1})+\lambda \sum_{j\;\neq \;k}\left|\sum_{jk}^{-1}\right|),$$

If the two samples are conditionally independent, the corresponding accuracy matrix coefficient is 0. At this time, $\sum^{-1}$ can be a positive semidefinite matrix. In order to find a sparse estimate of the inverse covariance matrix, the objective function can be simplified. The basic steps of applying the graphical lasso to dimensionality reduction in industrial systems are as follows:

Make

(3) $$S=\frac{1}{m}\sum_{i=1}^{m}{\left(x^{\left(i\right)}-\mu \right)}^{T}(x^{\left(i\right)}-\mu ),$$

Then

(4) $${\hat{\sum }}^{-1}={argmin}_{\sum^{-1}\geq 0}(tr(S\sum^{-1})-logdet(\sum^{-1})+\lambda \sum_{j\;\neq \;k}\left|\sum_{jk}^{-1}\right|),\;s.t.\;S\geq 0$$

where $\sum^{-1}$ is the inverse covariance matrix; 

S is the covariance matrix of the sample; 

λ is the regularization parameter.

This paper uses the graph lasso algorithm to solve the objective Function (4) and output the inverse covariance matrix as the result. The meaning of the zero elements in the inverse covariance matrix is that there is conditional independence between row-numbered variables and column-numbered variables. That is, if the elements in the i-th row and j-th column of $\sum^{-1}$ are 0, it means that given other variables, there is no relationship between the i-th variable and the j-th variable. Therefore, non-zero elements represent variables that exhibit high correlation under the influence of faults. The graphic lasso constructs an undirected graph model based on non-zero elements, where each process variable represents a node and the relationship between two process variables is represented by edges. Undirected graphs can assist researchers in identifying fault-related variables and effectively reduce the number of connections between variables.

### 3.2. False Causality Problem in Entropy Method

The sparse graph constructed by variable selection through a graphical lasso in industrial systems still involves multivariate scenarios. When conducting causal analysis on multivariate scenarios, the third condition for establishing causality must be considered. This condition states that the causal relationship between variables is uniquely determined by the interaction between two variables, independent of other factors. If the inferred causal relationships by causal analysis methods are not solely determined by the interaction between two variables but are influenced by misleading factors, this causal relationship is defined as spurious [26].

**Figure 1:**
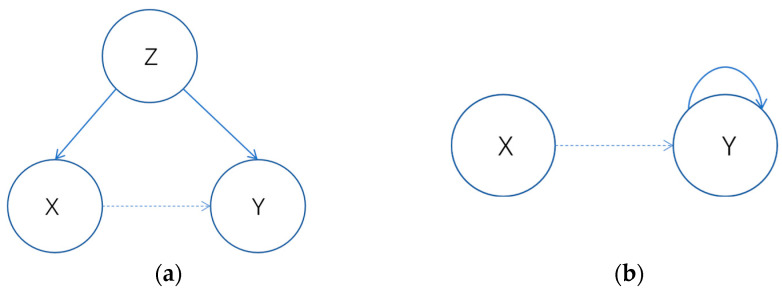
(**a**) Implicit variables lead to spurious causality; (**b**) autoregression leads to spurious causality.

In the context of industrial systems, the misleading influences on causal analysis can be analyzed from variable correlation and temporal characteristics. Firstly, due to the complex interconnections among variables in industrial systems, the variables selected for causal analysis may be jointly influenced by other variables (confounding variables). This can lead to spurious causal relationships between variables, where the relationships are modulated by the effects of confounding variables, showing common variations but lacking direct interactions. Secondly, the feedback control mechanisms in industrial systems introduce temporal characteristics to variables. The temporal nature of process variables can result in the current monitoring value of a variable being influenced by its past monitoring values, known as autoregression. In a multivariate system, the autoregression of process variables may mix with the effects of other variables, making it challenging to differentiate between causal relationships and the effects of autoregression. To avoid the emergence of spurious causal relationships in the analysis results, causal analysis methods should exclude the interference of autoregression and interactions with other confounding variables in the computation of variable pairs. Figure 1 provides a brief demonstration of the effects of autoregression and implicit variables

The calculation of transfer entropy results in biases caused by the aforementioned interference. This is because, according to the definition of transfer entropy, it measures not a specific value in a time series but all values in the sequence preceding the current time point. This definition necessitates estimating the problem of infinite dimensional density in calculating transfer entropy. In a straightforward transfer entropy calculation, tracing back in time from the current node must be truncated at a maximum value, denoted as $\tau_{max}$. Due to the temporal characteristics of variables leading to delayed effects, the commonly used fixed value of $\tau_{max}$ does not meet the practical requirements of dynamic industrial data, thereby impacting the accuracy of the analysis results [4].

**Figure 2:**
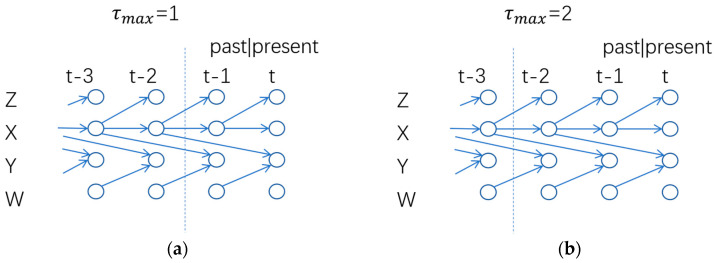
(**a**) $\tau_{max}$ takes 1 and the sequence on the right side of the dotted line to complete the calculation of transfer entropy and obtain the time series graph; (**b**) $\tau_{max}$ takes 2 and the sequence on the right side of the dotted line to complete the calculation of transfer entropy and obtain the time series graph.

Taking a multivariate Gaussian process model as an example, the influence of the cutoff value on the transfer entropy analysis results is analyzed:

(5a) $$Z_{t}=c_{XZ}X_{t-1}+\eta_{t}^{Z},$$

(5b) $$X_{t}=a_{X}X_{t-1}+\eta_{t}^{X}$$

(5c) $$Y_{t}=c_{XY}X_{t-2}+c_{WY}W_{t-1}+\eta_{t}^{Y}$$

(5d) $$W_{t}=\eta_{t}^{W}$$

represents Gaussian white noise, and the corresponding time series diagram is shown in Figure 2:

In a complete time series analysis, process the joint effects of process X and process W influence Y. When calculating the transfer entropy from process W to process Y, the influence of process X on process Y needs to be excluded. However, when $\tau_{max}$ is set to 1, the truncated time series reflects that process Y is only influenced by process W, while process X effectively becomes a confounding variable. This leads to an erroneous estimation of the strength of the influence of process W on process Y, ultimately affecting the results of causal analysis.

In the case where $\tau_{max}$ is set to 2, the influence of process X on process Y can be reflected in the truncated time series. However, since process X exhibits autoregression (where the current value of X is always related to the previous state), the calculation of transfer entropy from process X to process Y is contaminated by the internal dynamic effects of process X. This situation contradicts the scenario where the interactions between variables solely determine causality.

### 3.3. Causal Inference Based on LKIF

First consider a two-dimensional case:

(6) $$\frac{dx_{1}}{dt}=F_{1}\left(x_{1},x_{2},t\right),$$

(7) $$\frac{dx_{2}}{dt}=F_{2}\left(x_{1},x_{2},t\right).$$

This is a system of minimal dimensionality that admits information flow. Without loss of generality, examine only the flow/transfer from $x_{2}$ to $x_{1}$ [27].

Under the vector field, $F={\left(F_{1},F_{2}\right)}^{T}x$ evolves with time; correspondingly, its joint pdf ρ(x) evolves, observing a Liouville equation:

(8) $$\frac{\partial \rho }{\partial t}+\frac{\partial }{\partial x_{1}}(F_{1}\rho )+\frac{\partial }{\partial x_{2}}(F_{2}\rho )=0.$$

What matters here is the evolution of $H_{1}$, namely, the marginal entropy of $x_{1}$. For this purpose, integrate (8) with respect to $x_{2}$ over R to obtain

(9) $$\frac{\partial \rho_{1}}{\partial t}+\frac{\partial }{\partial x_{1}}\int_{R}(F_{1}\rho )dx_{2}=0.$$

Other terms vanish, thanks to the compact support assumption for ρ. Multiplication of (9) by $-(1+log\;\rho_{1})\;$followed by an integration over R gives the tendency of $H_{1}$:

(10) $$\frac{dH_{1}}{dt}=\int_{R^{2}}[log\rho_{1}\frac{\partial (F_{1}\rho )}{\partial x_{1}}]dx_{1}dx_{2}=-E(\frac{F_{1}}{\rho_{1}}\frac{\partial \rho_{1}}{\partial x_{1}}).$$

where E stands for mathematical expectation with respect to ρ. In the derivation, integration by parts has been used, as well as the compact support assumption.

#### 3.3.1. Excluding Autoregression

As the system guides the state forward, the marginal entropy of $x_{1}$ is replenished from two different sources: one from $x_{1}$ itself and the other from $x_{2}$. The latter is supplemented by the information flow/transmission mechanism itself. Decompose the increase in marginal entropy according to the underlying mechanism:

(11) $$\frac{dH_{1}}{dt}=\frac{dH_{1}^{*}}{dt}+T_{2\to 1}.$$

Liang and Kleeman believed that the change rate of marginal entropy $H_{1}$ caused only by $x_{1}$ should be

(12) $$\frac{dH_{1}^{*}}{dt}=\int_{R^{2}}\rho \frac{\partial F_{1}}{\partial x_{1}}dx_{1}dx_{2}.$$

This heuristic reasoning makes the separation (11) possible. Hence, the information flows from $x_{2}$ to $x_{1}$ at a rate of

(13) $$T_{2\to 1}=-\int_{R^{2}}\rho_{2|1}({x_{2}|x}_{1})\frac{\partial (F_{1}\rho_{1})}{\partial x_{1}}dx_{1}dx_{2}.$$

$\frac{dH_{1}^{*}}{dt}$ is the autoregressive effect of $x_{1}$, and $T_{2\to 1}$ is the information flow of $x_{2}$ to $x_{1}$. The Liang–Kleeman information flow calculation strictly distinguishes autoregression from information flow and eliminates the autoregressive effect.

#### 3.3.2. Eliminate Confounding Variables

For multivariate cases, take any variable pair i, j. Consider the information transfer rate from j to i [28]

(14) $$T_{j\to i}=\int_{R^{3}}\rho_{j|i}\left(x_{j}|x_{i}\right)\frac{\partial F_{i}\rho_{j}}{\partial x_{i}}dx-\frac{1}{2}\int_{R^{n}}\rho_{j|i}\left(x_{j}|x_{i}\right)\frac{\partial^{2}\left(b^{2}\rho_{j}\right)}{\partial x_{i}^{2}}dx.$$

The calculation formula does not contain any related operation terms of other variables, which can indicate that it is not affected by implicit variables.

Based on the above, the information flow calculation excludes the influence of autocorrelation and implicit variables, and the calculation results are unbiased, without false causal problems.

#### 3.3.3. Example

Starting from a simple case study, this section compares the information flow with the results of entropy calculation using current mainstream time series analysis methods. The system is a one-way coupled mapping from the literature [29]:

(15) $$X_{1,n+1}=f(X_{1,n}),$$

(16) $$X_{2,n+1}=(1-\varepsilon )f(X_{2,n})+\varepsilon g_{\alpha }(X_{1,n}).$$

According to [29]:

(17) f(x)=4x(1−x).

f(x) is a chaotic logic mapping, and $g_{\alpha }(x)$ a functional of f(x). Here we consider:

(18) $$g_{\alpha }(x)=\alpha f(x).$$

Select typical situations ε = 0.3, α = 1. The initialization sequence pairs are $X_{1,1}$ = 0.4, $X_{2,1}$ = 0.1.

The parameter selection is consistent with the recommended parameters in the transfer entropy literature for the transfer entropy method, with an autocorrelation coefficient of 4. The results are shown in Table 1 and saved in absolute value form:

LKIF and transfer entropy have the same method of judging the establishment of causal relationship, that is, the result obtained by the method is not 0. Generally, the causal strength threshold is set at 0.01. If this value is exceeded, the causal relationship is established.

For the given system, when α = 1, the system exhibits a one-way causal relationship from $X_{1}$ to $X_{2}$, where $T_{2\to 1}$ can be disregarded, $T_{1\to 2}$ being relatively significant, as expected based on the independence between $X_{1}$ and $X_{2}$. It can be confidently stated that the one-way causal relationships in the one-way coupled mappings, despite being highly nonlinear, have been somewhat well recovered through Equation (13).

In contrast, the computation results of transfer entropy reveal bidirectional causal relationships between $X_{1}$ and $X_{2}$, which undoubtedly deviate from the actual scenario.

Considering the above circumstances, utilizing information flow for causal analysis undoubtedly enhances the root cause analysis method’s capability for robust causal tracing.

### 3.4. Root Cause Analysis Process Analysis Based on LKIF–Glasso

**Figure 3:**
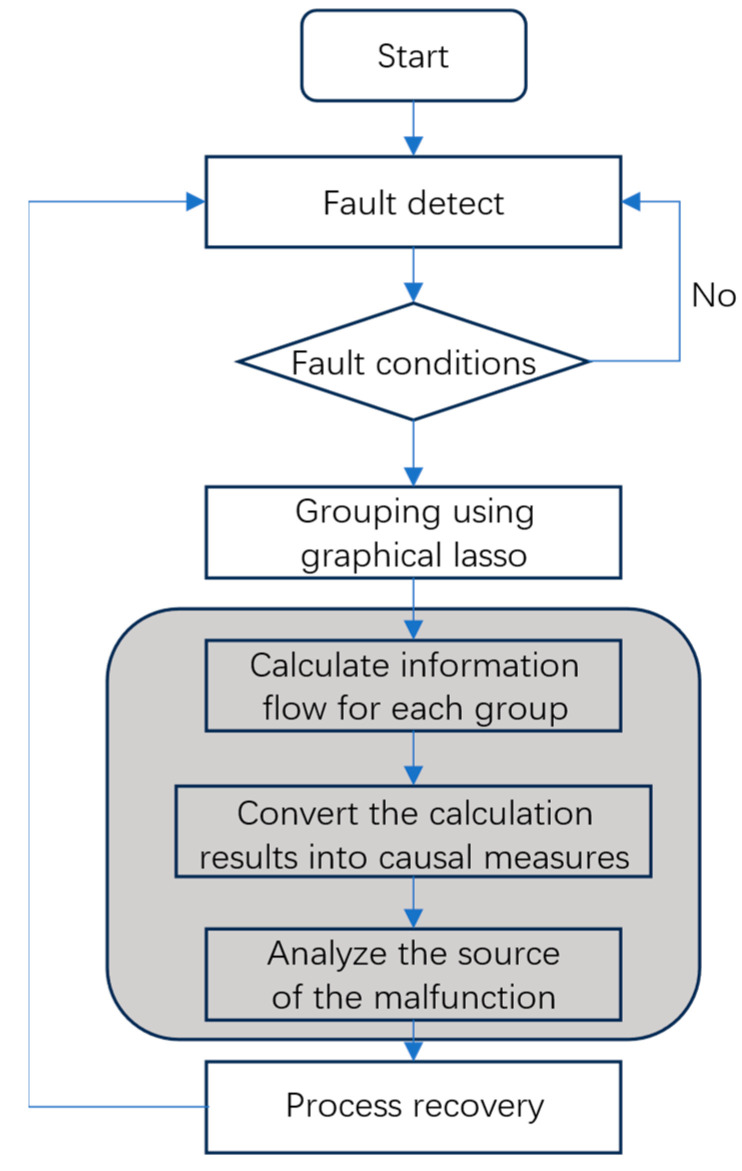
Flowchart of root cause analysis using the proposed method.

The proposed root cause analysis method is divided into two steps. First, the entire system variable set is grouped into smaller sets through the iterative graphical lasso algorithm, and the variable correlation graph is obtained. Then, the information flow intensity of the variable pair is calculated based on the standard operation data and the fault state data for the variables in the variable correlation graph. If the information flow is more significant than zero, it means the rate of change of the cause variable causes the entropy of the result variable to increase. If the information flow is less than zero, it means the rate of change of the cause variable causes the entropy of the result variable to decrease. According to the physical meaning of the information flow, the calculated information flow is converted into a causal measure, and the high-significance information flow is screened out to complete the fault traceability analysis. The gray box in Figure 3 represents the root cause analysis process

#### 3.4.1. Separation of Subgroups by Iterative Glasso

Input industrial operation data comes from process variables in various system links and consists of a set of time series. Graphical lasso usually causes a process variable to be deleted from the variable set, deleting all related variables. However, the correlation graph structure obtained based on the variable correlation measure does not mean the influence relationship and intensity between the variables. In order to avoid the deletion of key variables and key correlations, try to use the graphical lasso method in an iterative grouping manner. First, the required number of groups is determined empirically, and then the graphical lasso is applied to the entire set of monitored variables. After making the subsets, the graphical lasso is applied again to the remaining variables. Repeat this process until all variables are included in a group.

**Figure 4:**
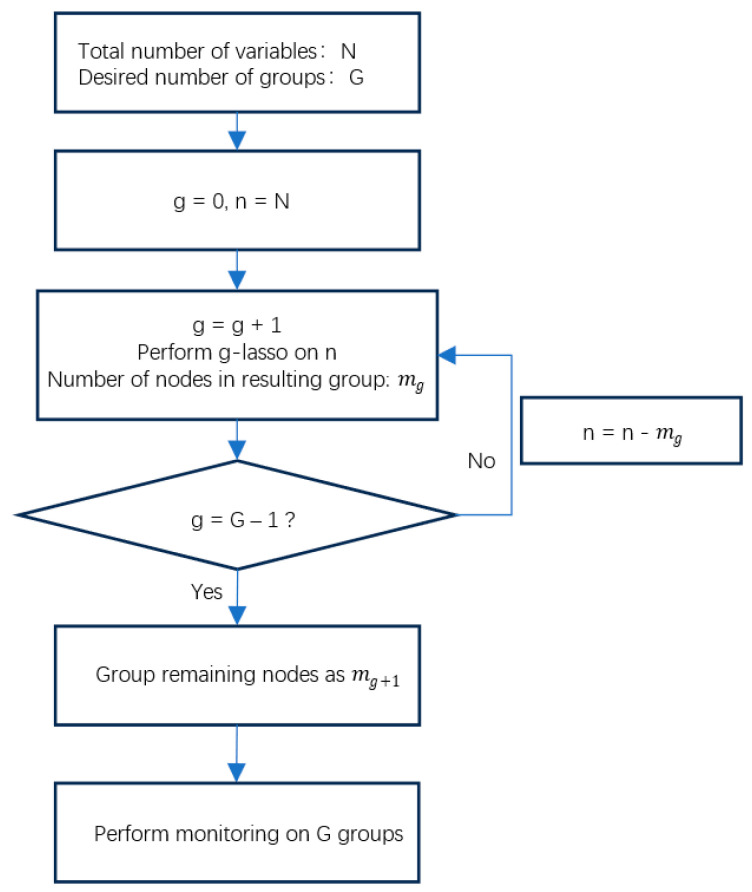
Iterative Glasso flowchart.

It should be noted that the value of the regularization parameter changes the structure of the generated undirected graph, which leads to the problem of selecting a suitable value. The goal of the graphical lasso is to obtain the structure of the undirected graph rather than to minimize the regression error like in lasso regression. Therefore, the value can be selectively determined according to its purpose of use [6]. Figure 4 shows the iterative process of the graphic lasso.

#### 3.4.2. Information Flow Computing

The information flow of each subcomponent is calculated to obtain measurements for causal relationship analysis. Calculating information flow involves comparing the difference in marginal entropy rate of considering and not considering the influence of causal variables. The net effect of the fault is obtained by subtracting the integrated information flow value during fault conditions from the integrated information flow value during the exact duration of the fault-free operation.

Because information flow represents the rate of change in the influence of cause variables on result variables, when the system is in a steady state, the rate of change is also in a steady state. However, when the system is affected by interference, the rate of change will also change with the change of the cause variable. In order to capture this change, we choose to use the coefficient of variation(CV) that can express the degree of data dispersion for preliminary screening [30].

(19) $$CV=\frac{STD(T_{j\to i})}{AVG(T_{j\to i})}.$$

Once the calculation of coefficient of variation within each subcomponent is completed, causal relationship measures can be derived for causal analysis. Information flow can have both positive and negative values. A negative value indicates that a process variable x leads to a more stable state change in process variable y. In contrast, a positive value indicates that a process variable x leads to a more chaotic state change in process variable y. Causal relationship measures with large positive or negative values indicate a significant causal influence of variable x on variable y. However, time series generated by chemical processes may be subject to noise interference. In particular, when $T_{2\to 1}$ = 0.653 ± 0.751 and $T_{1\to 2}$ = 1.240 ± 0.690, it is not sufficient to conclude that there is a unidirectional causal relationship from 1 to 2. In cases where the significance levels are close, the introduction of normalized information flow determines the fundamental causal variables [31].

The relative information flow is calculated as follows:

(20) $$Z_{2\to 1}=\left|T_{2\to 1}\right|+\left|\frac{dH_{1}^{*}}{dt}\right|+\left|\frac{dH_{1}^{noise}}{dt}\right|.$$

Here is the normalized information flow from $x_{2}$ to $x_{1}$:

(21) $$\tau_{2\to 1}=T_{2\to 1}/Z_{2\to 1}.$$

#### 3.4.3. Establishing LKIF–Glasso Root Cause Analysis Model

The main steps of the LKIF-Glasso root cause analysis model are as follows:

1. Input industrial operation data, which come from process variables at various stages of the system and are composed of a set of time series. One datum in the dataset represents a process variable. Then, construct a sequence table of process variables, which correspond one-to-one with the order of process variables in the dataset.
2. Use a graphical lasso to group the time series, remove highly correlated variables from the set, output the inverse covariance matrix, and construct a correlation graph based on the non-zero values of the variables represented by the matrix. Iterate step 2 until there are no variables in the set.
3. For the variables in each subgroup, calculate the instantaneous information flow intensity of the variables at each sampling time according to Formula (14), and represent the results in the form of a time series.
4. According to Formula (19), the coefficient of variation is obtained by taking the average and standard deviation of the information flow sequence to capture the step change when a fault occurs.
5. Based on the highly significant coefficient of variation, calculate the normalized information flow using Formula (21), construct the fault propagation path, and complete fault tracking.

## 4. Case Studies and Discussion

### 4.1. Case Study on the Tennessee Eastman Process

**Figure 5:**
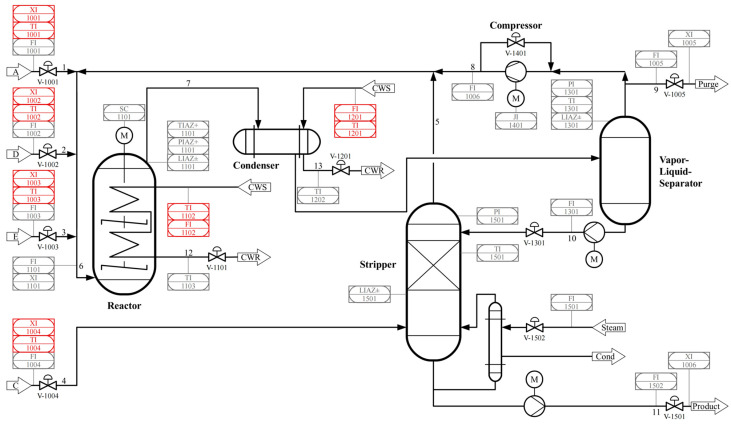
TE process flow diagram.

The Tennessee Eastman process is a classic benchmark for simulating industrial production processes. The TEP consists of five main units: a reactor, a condenser, a recycle compressor, a gas–liquid separator, and a stripping tower. Initially, the TEP version included 41 monitoring variables and 12 operable valves. The operating environment for TEP is MATLAB 2018a (MathWorks, Natick, MA, USA). The TEP model is depicted in the Figure 5 [32].

In the Tennessee Eastman process, there are four gas reactants, A, B, E, and C, each of which contains a small amount of inert gas B. Under the catalytic action, there are four simultaneous chemical reactions occurring in the reactor, with the two main reactions producing liquid products G and H. The chemical reaction equations are as follows:

(22) A(g)+C(g)+D(g)→G(liq),

(23) A(g)+C(g)+E(g)→H(liq),

It also includes two side chemical reactions as follows:

(24) A(g)+E(g)→F(liq),

(25) 3D(g)→2F(liq).

### 4.2. Experiment Plan

#### 4.2.1. Experimental Setup

The revised version of the Tennessee Eastman process includes 28 types of faults. The specific descriptions of the fault-related issues are outlined in Table 2. Among these, faults related to the stripping tower have the most significant impact on the quality of chemical process products due to their involvement in the separation of raw materials, intermediate products, and final products. Therefore, this study selects IDV(7) related to the stripping tower (C header pressure loss—reduced availability) as the research subject for detailed discussion. At the end of this chapter, the root cause analysis results of the LKIF Glasso method for all TEP faults are provided. The parameters related to dataset generation are detailed in Table 3.

In TEP, 41 monitored variables and 12 operable valves are represented as process variables. Out of a total of 53 process variables, 50 variables were analyzed, excluding the three manipulated variables (compressor cycle valve, stripper steam valve, and agitator speed) with fixed values under MultiLoop_mode1 conditions. The correspondence between process variables and actual physical quantities is shown in Table 4.

#### 4.2.2. Comparison Methods and Parameter Selection

In order to verify the performance and improvement effect of the proposed fault tracking method, this chapter will compare the results of the proposed method with the transfer entropy method, which is also based on entropy.

For the parameter settings of the transfer entropy method, please refer to Brian Lindner [20], as shown in Table 5.

### 4.3. Experimental Result

To implement the proposed method, the 50 variables of the TEP were divided into five subgroups using graphical lasso, and information flow calculations were performed for each subgroup. The hyperparameters of graphical lasso were adjusted to roughly include ten process variables in each subgroup. The subgroup separation results are shown in Table 6.

#### 4.3.1. LKIF Calculation Results

**Figure 6:**
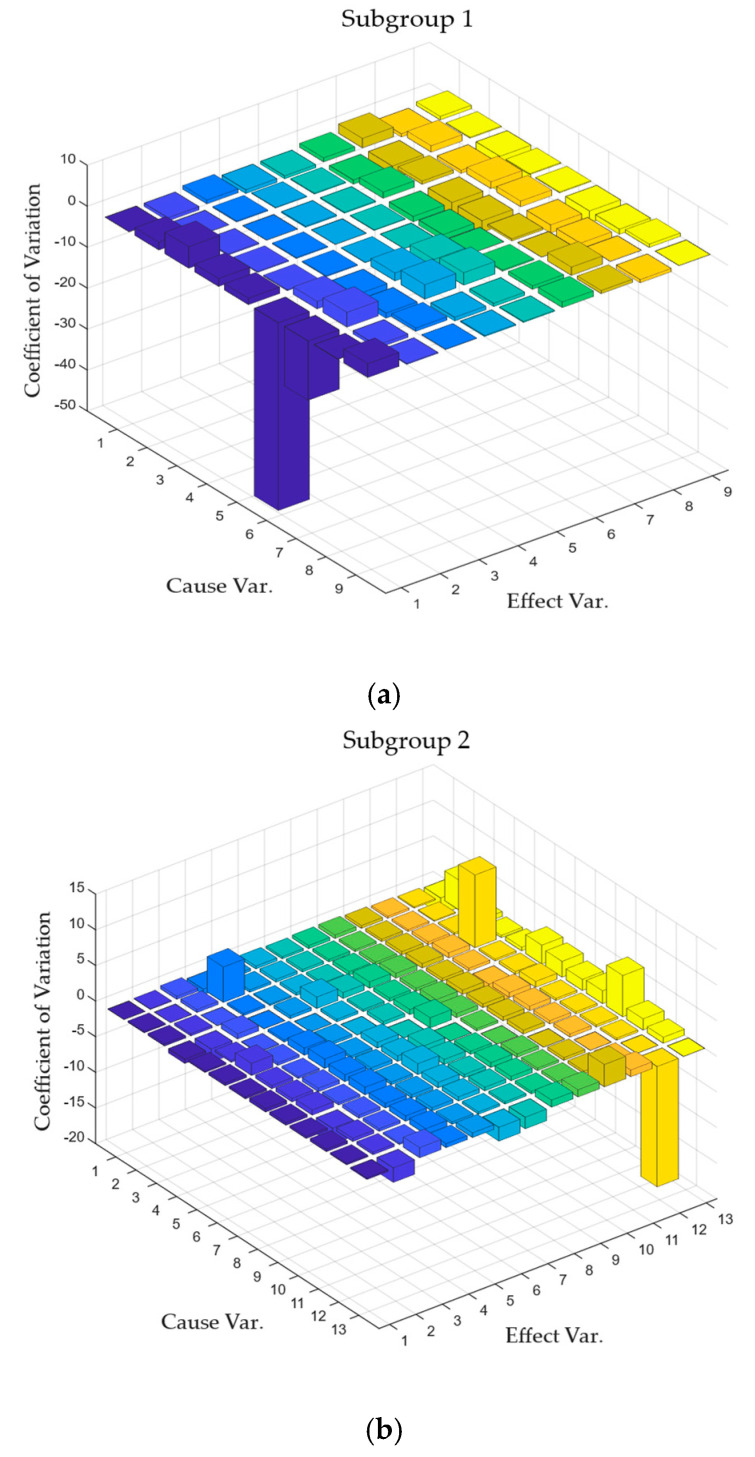

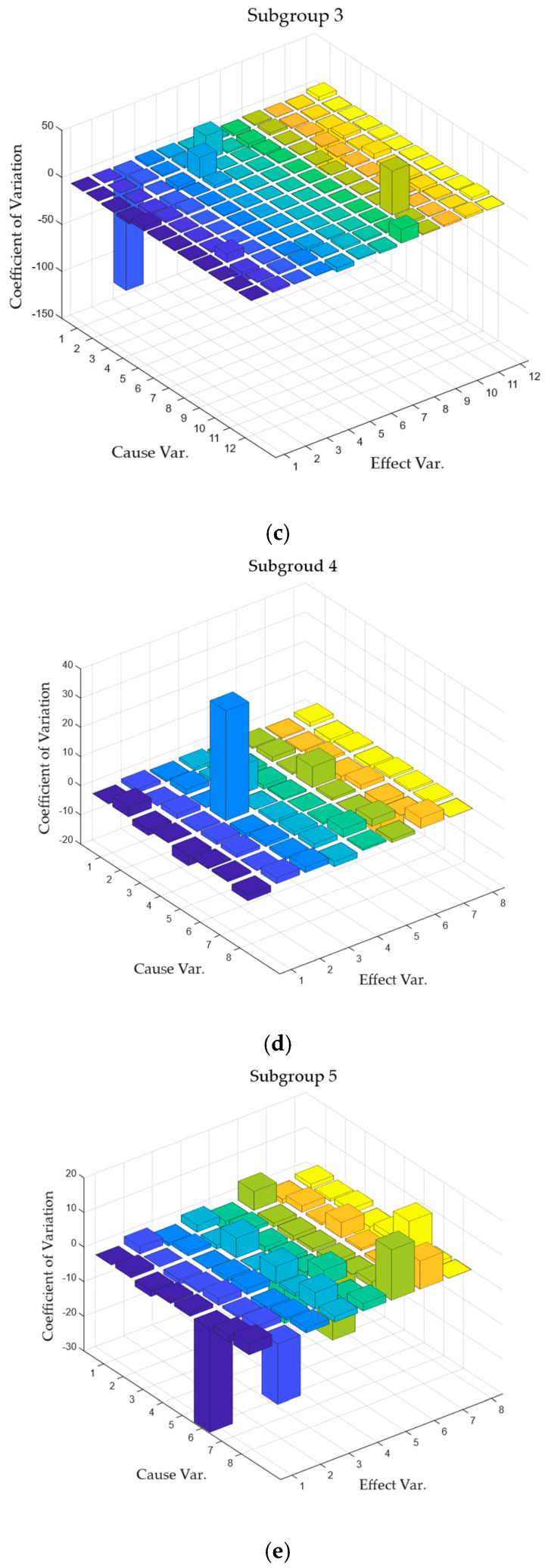
Coefficient of variation plot of IDV (7) in each subgroup. The process variables corresponding to the subgroup numbers in the figure correspond to the sorting of subgroup members in Table 7: (**a**) subgroup 1; (**b**) subgroup 2; (**c**) subgroup 3; (**d**) subgroup 4; (**e**) subgroup 5.

Based on the subgroup separation results, information flow calculations were performed pairwise for the process variables within each subgroup. Since information flow calculates the instantaneous state of information transfer, process variables were influenced by introduced disturbances, leading to transitions in the information flow between process variables. To measure the magnitude of these transitions in information flow, the coefficient of variation, which represents the degree of data dispersion, was chosen as a metric for initial inference. The calculation results are shown in Figure 6.

**Figure 7:**
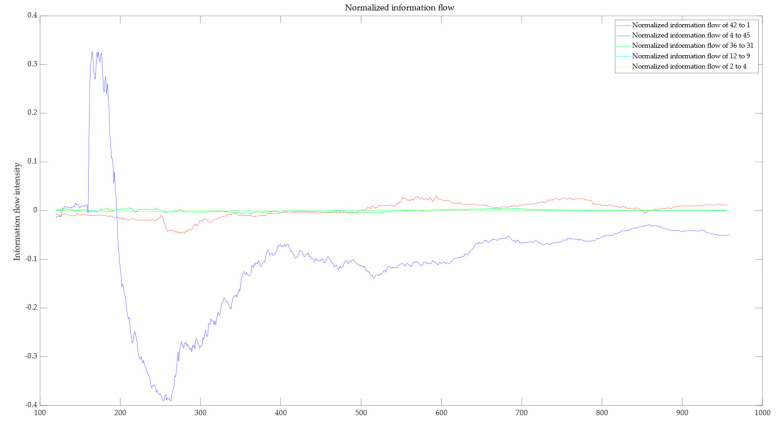
Normalized information flow.

The results of coefficient of variation calculations for each subgroup were summarized and sorted. The top five significant coefficient of variation values were selected, and the corresponding process variables were defined as potential root cause variables. The screening results are shown in Table 7. Due to cases where a transition occurs from an information flow close to zero to another close to zero but with significantly high coefficient of variation, it is necessary to further calculate the normalized information flow for the potential root cause variables. Based on the values of the information flow, the true root cause variables were determined. The results of the normalized information flow calculations are shown in Figure 7.

#### 4.3.2. Transfer Entropy Calculation Results

**Figure 8:**
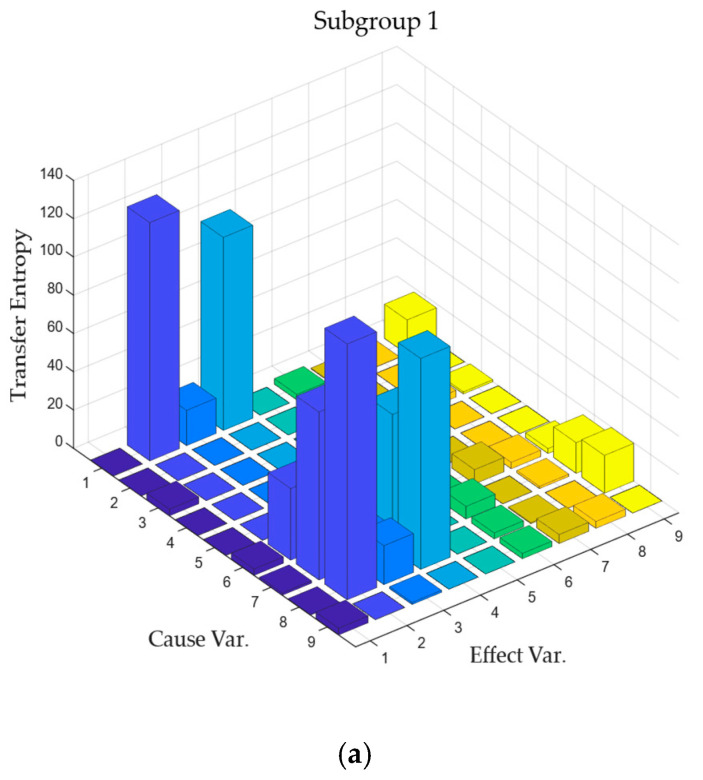

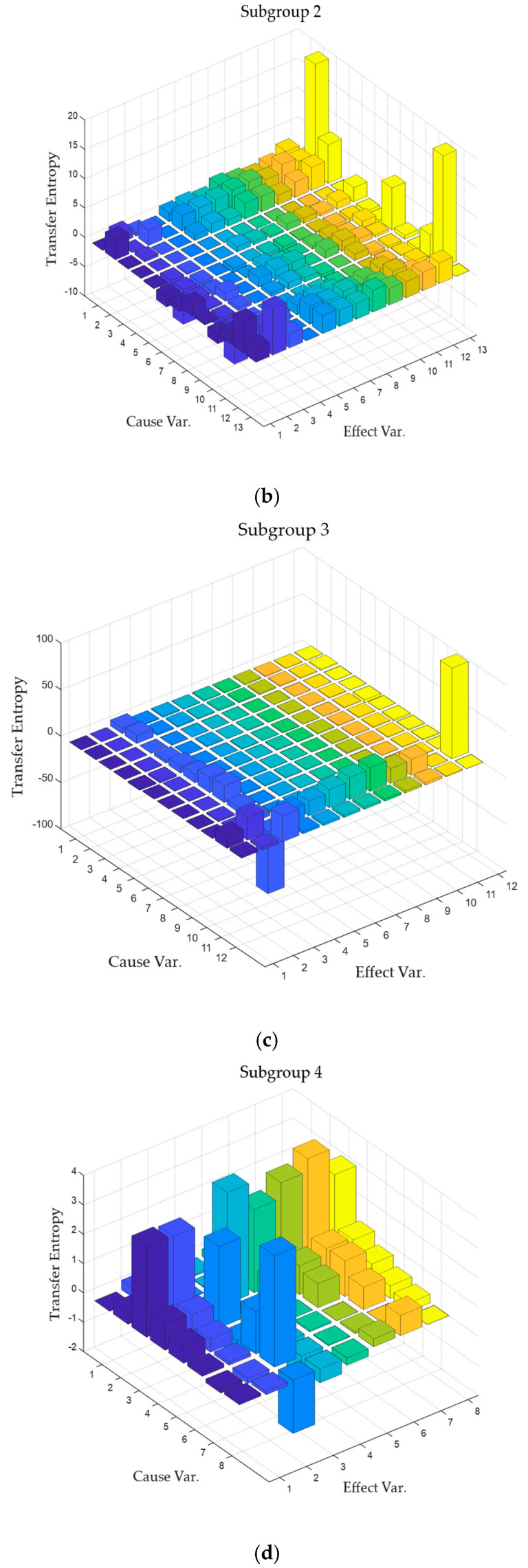

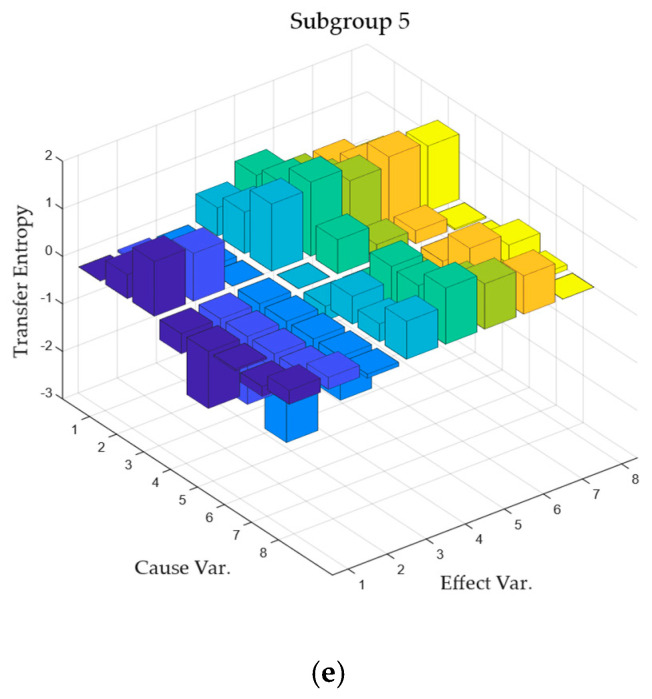
Transfer entropy plot of IDV (7) in each subgroup. The process variables corresponding to the subgroup numbers in the figure correspond to the sorting of subgroup members in Table 7: (**a**) subgroup 1; (**b**) subgroup 2; (**c**) subgroup 3; (**d**) subgroup 4; (**e**) subgroup 5.

Consistent with LKIF, pairwise transfer entropy calculations were performed for the process variables within each subgroup based on the subgroup separation results. The calculation results of transfer entropy are shown in Figure 8.

According to the causal analysis process proposed by Lee et al. [23], all calculation results were normalized and a significance threshold of 0.85 was set. Variables that exceeded the threshold were screened and are represented in Table 8.

### 4.4. Discussion of Results

It is not difficult to see from Figure 7 that among the five pairs of process variables with the highest coefficient of variation, except for the normalized information flow between process variables 4 and 45, the normalized information flow of other variable pairs tends to zero. In the definition of information flow, a calculated information flow close to zero means that there is no causal relationship between variable pairs. Therefore, the basic causal variable obtained through the LKIF–Glasso method is process variable 4.

To reinforce this result, process variable 4 was used as the fault source and fault propagation was derived. The derivation process was based on chemical engineering principles to predict changes in TEP monitoring variables and compare them with actual observed values.

The cause of fault seven can be clearly determined: when the total feed flow rate is running, the process variables of reactor pressure and product separation pressure changes are measured separately. Components A, C, D, and E participate in the reaction and are in the gas phase. When the pressure of component C decreases, in order to compensate for the pressure drop in the C reflux line, the feed flow rate of component AC is increased by adjusting the flow valve on flow rate 4. The feed rates of components A and C decrease, and in order to maintain a stable feed ratio, the feed rate of component A increases. Due to the influence of the exchange feed flow rate and A feed flow rate, the total feed flow rate and reactor liquid level decrease.

In addition, the gas phase inside the reactor affects the reactor pressure. Therefore, the pressure decreases, and due to the connection of the reactor to the separator and stripping tower, the pressure in the separator and stripping tower also decreases. In the stripping tower, the pressure drops and the change in pressure in the C reflux pipeline disrupts the gas–liquid equilibrium, ultimately leading to the failure of the stripping tower and excessive temperature [32].

**Figure 9:**
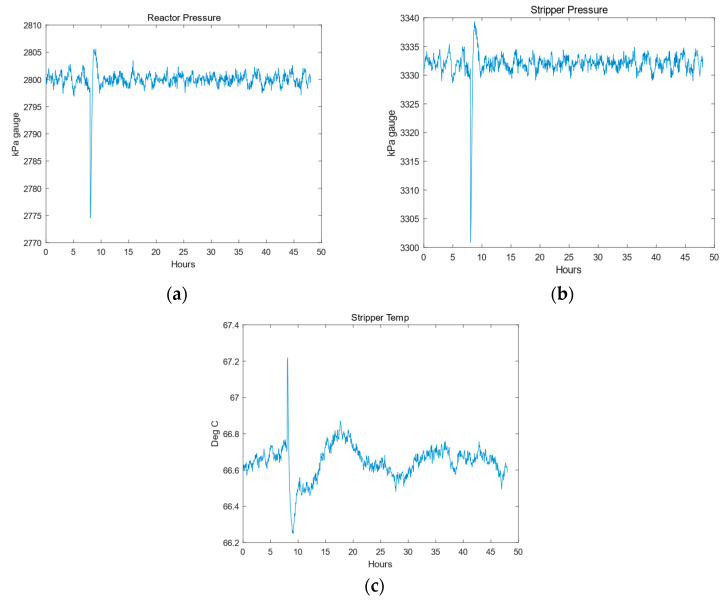
Actual changes in monitoring variables in TEP: (**a**) reactor pressure; (**b**) stripper pressure; (**c**) stripper temp.

By comparing the derivation process with the actual changes recorded truthfully in Figure 9, process variable 4 was ultimately confirmed as the root cause variable, consistent with the results published in Table 1. These documents also provide strong support [33,34,35].

The most significant variable pairs obtained by entropy transfer calculation are 44 pairs of seven, 1 pair of seven, and 45 pairs of four. Among them, process variable 44 and process variable 1 correspond to the feeding situation of component A. It is not difficult to deduce from the above chemical principles that the change in the feeding situation of component A is actually indirectly caused by the pressure loss of component C. However, the calculation results of the transfer entropy believe that the change in component A itself is the root cause, which is not in line with the chemical principles. At the same time, it is also inconsistent with the root cause of fault 7 disclosed in Table 1. It can be said that the root cause analysis results of the transfer entropy are erroneous. In the end, a false root cause was obtained.

Finally, the inference results of the LKIF–Glasso method for all 28 TEP faults are shown in Table 9.

## 5. Conclusions

This article proposes the LKIF–Glasso method to improve the performance of entropy-based causal analysis in chemical fault root cause analysis. The LKIF–Glasso method divides root cause analysis into two steps: dimensionality reduction and traceability. Firstly, the industrial operation data are dimensionality reduced through graphical lasso. Secondly, the LKIF method is used to measure the causal effects of the subgroups separated by the graphical lasso.

The graphic lasso should address the high dimensionality of industrial systems by extracting correlations between variables and screening out fault related variables, ensuring the timeliness and accuracy of root cause analysis. In the experiment, the initial state requires calculating the information flow intensity of 2500 variable pairs, while using a graphical lasso only requires calculating the information flow intensity of 522 variable pairs.

The LKIF method was used to address the issue of false causality in the transfer entropy method. A comparison was made between fault 7 in the TEP and the transfer entropy method. The calculation results of the two methods showed that LKIF could calculate the root cause without deviation, while the transfer entropy showed deviation. And at the end, all 28 faults in the TEP were traced to demonstrate that the proposed method is applicable to multiple faults.

The application of entropy in the field of fault tracing provides a scientific basis for revealing the essence of faults, locating the source of faults, and optimizing fault handling processes. With the development of entropy theory and related technologies, the application of entropy in the field of fault tracing will become more profound, which will help improve the safety and reliability of industrial systems and reduce the economic losses caused by faults.

## Figures and Tables

**Table 1:** Information flow and transfer entropy result comparison.

Strength of Causal Impact	Liang-Kleeman IF	Transfer Entropy
$X_{2}$ to $X_{1}$	0.0075	1.6979
$X_{1}$ to $X_{2}$	0.1880	2.4411

**Table 2:** Process faults in Tennessee Eastman Process (TEP).

No.	Description (Root-Cause Variable)	Type
IDV(1)	A/C feed ratio, B composition constant (stream 4)	Step
IDV(2)	B composition, A/C ratio constant (stream 4)	Step
IDV(3)	D feed temperature (stream 2)	Step
IDV(4)	Reactor cooling water inlet temperature	Step
IDV(5)	Condenser cooling water inlet temperature	Step
IDV(6)	A feed loss (stream 1)	Step
IDV(7)	C header pressure loss—reduced availability (stream 4)	Step
IDV(8)	A, B, C feed composition (stream 4)	Random variation
IDV(9)	D feed temperature (stream 2)	Random variation
IDV(10)	C feed temperature (stream 4)	Random variation
IDV(11)	Reactor cooling water inlet temperature	Random variation
IDV(12)	Condenser cooling water inlet temperature	Random variation
IDV(13)	Reaction kinetics	Slow drift
IDV(14)	Reactor cooling water valve	Sticking
IDV(15)	Condenser cooling water valve	Sticking
IDV(16)	Unknown ^1^	Random variation
IDV(17)	Unknown ^1^	Random variation
IDV(18)	Unknown ^1^	Random variation
IDV(19)	Unknown ^1^	Sticking
IDV(20)	Unknown ^1^	Unknown ^1^
IDV(21)	A feed temperature (stream 1)	Random variation
IDV(22)	E feed temperature (stream 3)	Random variation
IDV(23)	A feed pressure (stream 1)	Random variation
IDV(24)	D feed pressure (stream 2)	Random variation
IDV(25)	E feed pressure (stream 3)	Random variation
IDV(26)	A and C feed pressure (stream 4)	Random variation
IDV(27)	Reactor cooling water pressure	Random variation
IDV(28)	Condenser cooling water pressure	Random variation

^1^ Unknown: uncovered by A. Bathelt in revised MATLAB version model.

**Table 3:** Generate relevant parameters for the dataset used in the experiment.

Parameter	Value
TEP_mode	MultiLoop_mode1
Simulation total duration	48 h
Interference introduction time	8 h
Type of interference introduction	IDV(7)
Sampling frequency	20 samples per hour

**Table 4:** Process variables of TEP.

Variable No.	Variable Name	Variable No.	Variable Name
1	A feed flowrate (stream 1)	18	Stripper temperature
2	D feed flowrate (stream 2)	19	Stripper steam flowrate
3	E feed flowrate (stream 3)	20	Compressor work
4	A and C feed flowrate (stream 4)	21	Reactor c/w outlet temperature
5	Recycle flowrate (stream 8)	22	Separator c/w outlet temperature
6	Reactor feed rate (stream 6)	23–28	Reactor feed analysis (A-F mol%) (stream 6)
7	Reactor pressure	29–36	Purge gas analysis (A-H mol%) (stream 9)
8	Reactor level	37–41	Product analysis (D-H mol%) (stream 11)
9	Reactor temperature	42	D feed flow valve (stream 2)
10	Purge rate (stream 9)	43	E feed flow valve (stream 3)
11	Product separator temperature	44	A feed flow valve (stream 1)
12	Product separator level	45	A and C feed flow valve (stream 4)
13	Product separator pressure	46	Purge valve (stream 9)
14	Product separator under flowrate (stream 10)	47	Separator pot liquid flow valve (stream 10)
15	Stripper level	48	Stripper liquid product flow valve (stream 11)
16	Stripper pressure	49	Reactor c/w flow rate
17	Stripper under flowrate (stream 11)	50	Condenser c/w flow rate

**Table 5:** Hyperparameters of transfer entropy in TEP.

Hyperparameters	Value
Embedding dimension of cause variable (L)	4
Embedding dimension of cause variable (K)	1
Prediction horizon (H)	4
Estimated delay (τ=H)	4

**Table 6:** Subgroup separation results.

Subgroup No.	Subgroup Members
1	1, 7, 10, 13, 16, 42, 43, 44, 46
2	11, 18, 20, 21, 22, 23, 29, 30, 34, 35, 38, 47, 49
3	2, 3, 4, 15, 24, 25, 27, 28, 31, 36, 45, 48
4	6, 8, 9, 12, 14, 33, 37, 50
5	5, 17, 19, 26, 32, 39, 40, 41

**Table 7:** The top five significant coefficient of variation values.

Process Variable Pair	Coefficient of Variation
2 to 4	−123.1235
4 to 45	−48.7168
36 to 31	46.8663
42 to 1	−45.8058
12 to 9	37.6357

**Table 8:** Normalization result of entropy transfer.

Process Variable Pair	Normalization Results
44 to 7	1
1 to 7	0.9083
45 to 4	−1

**Table 9:** Diagnostic results and analysis of all faults in TEP.

No.	Diagnosis Result of the Proposed Methodology (Variable No.)
IDV(1)	38, 47
IDV(2)	10
IDV(3)	43, 44, 46
IDV(4)	49
IDV(5)	18
IDV(6)	44
IDV(7)	4
IDV(8)	20, 47
IDV(9)	18
IDV(10)	18
IDV(11)	49
IDV(12)	16, 21
IDV(13)	21, 47
IDV(14)	9
IDV(15)	44
IDV(16)	18
IDV(17)	9, 21
IDV(18)	22
IDV(19)	45, 48
IDV(20)	21, 47
IDV(21)	13
IDV(22)	16
IDV(23)	44
IDV(24)	21
IDV(25)	44, 47
IDV(26)	45
IDV(27)	49
IDV(28)	18

## Data Availability

Data is contained within the article.

## References

[1] Cheng Y., Wang L., Zhao X. (2023). A Review of Root Cause Analysis Research. Res. Comput. Appl..

[2] Qi C. (2023). Causal Analysis Method for Industrial Process Operation Faults Based on Transfer Entropy.

[3] Schreiber T. (2000). Measuring Information Transfer. Phys. Rev. Lett..

[4] Runge J., Heitzig J., Marwan N., Kurths J. (2012). Quantifying Causal Coupling Strength: A Lag-specific Measure for Multivariate Time Series Related to Transfer Entropy. Phys. Rev. E Stat. Nonlinear Soft Matter Phys..

[5] Huang J., Kuang Z., Ma J., Fang Y. (2024). Performance prediction method based on Liang-Kleeman information flow and wood tracheid morphology. Mater. Today Commun..

[6] Kim C., Lee H., Kim K., Lee Y., Lee W.B. (2018). Efficient Process Monitoring via the Integrated Use of Markov Random Fields Learning and the Graphical Lasso. Ind. Eng. Chem. Res..

[7] Friedman J., Hastie T., Tibshirani R. (2008). Sparse inverse covariance estimation with the graphical lasso. Biostatistics.

[8] Ji C. (2023). Research on Data Based Non Stationary Chemical Process Monitoring Method.

[9] Yang B., Xiong Y., Fu L., Xu W., Li J. (2024). Industrial digital transformation: Research progress on fault diagnosis methods. Big Data.

[10] Felix L.O., de Sá Só Martins D.H.C., Monteiro U.A.B.V., Pinto L.A.V., Tarrataca L., Martins C.A.O. (2024). Multiple Fault Diagnosis in a Wind Turbine Gearbox with Autoencoder Data Augmentation and KPCA Dimension Reduction. J. Nondestruct. Eval..

[11] Harkat M.F., Mansouri M., Nounou M.N., Nounou H.N. (2019). Fault detection of uncertain chemical processes using interval partial least squares-based generalized likelihood ratio test. Inf. Sci..

[12] Khurshid A., Pani K.A. (2024). An integrated approach combining randomized kernel PCA, Gaussian mixture modeling, and ICA for fault detection in nonlinear processes. Meas. Sci. Technol..

[13] Jiang J., Chen R., Zhang C., Chen M., Li X., Ma G. (2020). Dynamic Fault Prediction of Power Transformers Based on Lasso Regression and Change Point Detection by Dissolved Gas Analysis. IEEE Trans. Dielectr. Electr. Insul..

[14] Zheng K., Li T., Su Z., Zhang B. (2020). Sparse Elitist Group Lasso Denoising in Frequency Domain for Bearing Fault Diagnosis. IEEE Trans. Ind. Inform..

[15] Zhang H., Peng K., Ma L. (2023). A systematic nonstationary causality analysis framework for root cause diagnosis of faults in manufacturing processes. Control Eng. Pract..

[16] Interateneo D. (2008). Kernel method for nonlinear Granger causality. Phys. Rev. Lett..

[17] Sugihara G., May R., Ye H., Hsieh C.H., Deyle E., Fogarty M., Munch S. (2012). Detecting Causality in Complex Ecosystems. Science.

[18] Ye H., Deyle E.R., Gilarranz L.J., Sugihara G. (2015). Distinguishing time-delayed causal interactions using convergent cross mapping. Sci. Rep..

[19] Hu W., Wang J., Chen T., Shah S.L. (2017). Cause-effect analysis of industrial alarm variables using transfer entropies. Control Eng. Pract..

[20] Lindner B., Auret L., Bauer M. (2019). A Systematic Workflow for Oscillation Diagnosis Using Transfer Entropy. IEEE Trans. Control Syst. Technol..

[21] Jizba P., Korbel J. (2014). Multifractal Diffusion Entropy Analysis: Optimal Bin Width of Probability Histograms. Phys. A Stat. Mech. Its Appl..

[22] Tang Q. (2020). Research on Data-Driven Industrial Process Fault Detection and Identification.

[23] Lee H., Kim C., Lim S., Lee J.M. (2020). Data-Driven Fault Diagnosis for Chemical Processes Using Transfer Entropy and Graphical Lasso. Comput. Chem. Eng..

[24] Zhang L., Liao X., Dong P., Hou S., Li B., Chen Z. (2024). An Efficient Method for Identifying Inter-Well Connectivity Using AP Clustering and Graphical Lasso: Validation with Tracer Test Results. Processes.

[25] Dallakyan A. (2022). Graphiclasso: Graphical lasso for learning sparse inverse-covariance matrices. Stata J..

[26] Yao L., Chu Z., Li S., Li Y., Gao J., Zhang A. (2021). A Survey on Causal Inference. ACM Trans. Knowl. Discov. Data.

[27] Liang X.S., Kleeman R. (2005). Information transfer between dynamical system components. Phys. Rev. Lett..

[28] Liang X.S. (2015). Information flow and causality as rigorous notions ab initio. Phys. Rev. E.

[29] Runge J., Petoukhov V., Kurths J. (2014). Quantifying the Strength and Delay of Climatic Interactions: The Ambiguities of Cross Correlation and a Novel Measure Based on Graphical Models. J. Clim..

[30] Huang J., Liu J., Liu H., Yang X., Ma J. Research on nuclear power fault location method based on info-flow SDG model. Proceedings of the Asia Conference on Electrical, Power and Computer Engineering.

[31] Liang X. (2013). The Liang-Kleeman Information Flow: Theory and Applications. Entropy.

[32] Bathelt A., Ricker N.L., Jelali M. Revision of the Tennessee Eastman Process Model. Proceedings of the IFAC International Symposium on Advanced Control of Chemical Processes.

[33] Bi X., Wu D., Xie D., Ye H., Zhao J. (2023). Large-scale chemical process causal discovery from big data with transformer-based deep learning. Process Saf. Environ. Prot..

[34] Zhang Y., Zhang S., Jia X., Zhang X., Tian W. (2023). A novel integrated fault diagnosis method of chemical processes based on deep learning and information propagation hysteresis analysis. J. Taiwan Inst. Chem. Eng..

[35] Zhang J., Luo W., Dai Y., Yao Y. (2022). Cycle temporal algorithm-based multivariate statistical methods for fault diagnosis in chemical processes. Chin. J. Chem. Eng..

